# A second course of antithyroid drug therapy for recurrent Graves' disease: an experience in endocrine practice

**DOI:** 10.1530/EJE-14-0704

**Published:** 2015-03

**Authors:** Xiaomei Liu, Wei Qiang, Xingjun Liu, Lianye Liu, Shu Liu, Aibo Gao, Shan Gao, Bingyin Shi

**Affiliations:** Department of Endocrinology, First Affiliated Hospital of Xi'an Jiaotong University, No. 277 Yanta West Road, Xi'an, Shaanxi, 710061, China

## Abstract

**Objective:**

There are scarce reports regarding the prognosis of a second course of antithyroid drug (ATD) therapy on recurrent Graves' disease (GD). The aim of this study was to assess the long-term remission rate after a second ATD therapy and verify significant clinical predictors of a remission.

**Design:**

A prospective randomized clinical trial with long-term follow-up was conducted to evaluate the effects of a second course of ATD therapy.

**Methods:**

A total of 128 recurrent GD patients who had finished a first regular ATD therapy were enrolled in this study, and prescribed methimazole (MMI) treatment with titration regimen. The patients were randomly assigned to two groups when the drug doses were reduced to 2.5 mg daily (qd). Group 1 was discontinued with 2.5 mg qd after about 5 months. Group 2 was continuously reduced to 2.5 mg every other day (qod) after 5 months and then discontinued with 2.5 mg qod after about a further 5 months. The patients were followed for 48 months after drug withdrawal.

**Results:**

Of the total number of patients, 97 cases (75.78%) achieved permanent remission at the end of follow-up, with the recurrence of 31 cases (24.22%). The remission rate of group 2 (84.62%) was significantly higher than that of group 1 (66.67%) (*P*=0.024). Cox regression showed that the hazard ratio for recurrence decreased under a high or high normal TSH level at drug withdrawal.

**Conclusion:**

A second course of ATD therapy can bring about a satisfying long-term remission on recurrent GD. The drug dose of 2.5 mg qod and a high or high normal TSH level at drug withdrawal may increase the likelihood of permanent remission.

## Introduction

Antithyroid drug (ATD) is one of the main therapeutic modalities for Graves' disease (GD) (1). One major problem with ATD therapy is the high recurrence rate, which is estimated to be 40% (2) or even 50–60% (3). Thus, the treatment strategy for recurrent GD is extremely urgent. Some clinicians affirmed that if hyperthyroidism recurs after ATD therapy, there is little chance that a second course of ATD treatment will result in permanent remission, and they recommend radioactive iodine for patients with recurrent hyperthyroidism, unless there is an indication for surgery (4). Cooper (5) concluded that definitive radioiodine therapy is the preferred strategy in adults who have a relapse, while ATD is preferable for children and adolescents in recurrent GD. It was also pointed out that prolonged low-dose ATD therapy may be feasible in patients with previous relapse who prefer drug therapy rather than surgery or radioiodine (6). However, there are few prospective clinical trials evaluating ATD therapy in recurrent GD and it is uncertain whether ATD therapy will prevent the permanent remission of recurrent hyperthyroidism. This study was carried out to evaluate the long-term efficiency and prognosis of ATD therapy with different discontinuation doses of methimazole (MMI) in Chinese patients with recurrent GD.

## Subjects and methods

### Experimental design

A prospective clinical study was implemented to investigate the long-term prognosis of ATD therapy on recurrent GD, with two different discontinuation doses of MMI, in the department of Endocrinology, First Affiliated Hospital of Xi'an Jiaotong University. The consent was obtained from each patient after full explanation of the purpose and nature of all procedures used. All of the enrolled patients agreed to join this clinical trial and signed the informed consent. The protocol was approved by the local ethical committee, functioning according to the third edition of the Guidelines on the Practice of Ethical Committees in Medical Research issued by the Royal College of Physicians of London. The clinical trial registration number is ChiCTR-ONRC-13003306.

### Inclusion and exclusion criteria

Diagnosis of recurrent GD was made upon the patient's GD history with a finished regular ATD therapy, the clinical features of GD recurrence including the presence of conventional symptoms of hyperthyroidism associated with a diffusely enlarged goiter, elevated levels of free tri-iodothyronine and free thyroxine, and decreased thyroid-stimulating hormone (TSH) level. All enrolled patients were first diagnosed as recurrent GD and none of the patients had been treated for recurrent GD before inclusion.

Exclusion criteria were i) pregnant or lactating women, ii) patients combined with thyroid tumor or thyroiditis, and iii) serious hepatic impairment.

### Patients

A total of 143 consecutive patients with recurrent GD were prospectively enrolled in this clinical trial. Of them, 15 patients were excluded from the study till the end of the follow-up due to the following reasons: six patients dropped out of the study (three were pregnant and switched to propylthiouracil, one underwent surgery, and two accepted radioiodine therapy) and the remaining nine patients were lost. Thus, 128 patients (107 females, 21 males, age 34.88±12.58 years, mean±s.d.) were retained for analysis.

### Therapy

After informed consent, the enrolled patients were prescribed a daily starting dose of 7.5–30 mg MMI (mean±s.d., 15.06±7.69) according to the severity of symptoms and thyroid function, and followed up by the titration regimen (30 mg→20 mg→15 mg→10 mg→7.5 mg→5 mg→2.5 mg) (where the ATD dose is adjusted gradually according to thyroid hormone concentrations). When the daily dose was reduced to 2.5 mg qd with normal thyroid functions, the patients would be assigned by the random number table to group 1 (stop the drug therapy after 5 months of 2.5 mg qd with normal thyroid functions, or stop after occurrences of clinical or subclinical hypothyroidism during the 5 months) or group 2 (reduce the drug dose to 2.5 mg qod after 2.5 mg qd in 5 months, and then discontinue after 5 months of 2.5 mg qod with normal thyroid functions, or discontinue after occurrences of clinical or subclinical hypothyroidism during the further 5 months). With the initial large drug dose, the follow-up interval was ∼1 month or so. Then, the intervals could be prolonged with dose reduction, and when the MMI was reduced to 5 mg qd or less, the intervals can be increased to every 4–6 months.

The follow-up was carried out continuously for 1, 3, 6, 9, 12, 18, 24, 36 and 48 months after drug withdrawal, and the thyroid functions were recorded.

### Statistical analysis

The data were analyzed using an SPSS software program (version 18.0; SPSS, Inc.). Values of *P*<0.05 were considered to indicate statistical significance. A *χ* ^2^-test was used to compare the qualitative variables. Quantitative variables were analyzed by the Student's *t*-test (normal distribution) or Mann–Whitney *U* test (abnormal distribution). The risk of relapse was evaluated using Kaplan–Meier survival analysis between groups, and Cox regression analysis was also used to evaluate prognostic factors.

## Results

### Clinical and biological characteristics

The baseline characteristics and some parameters at drug withdrawal are given in Table 1. Group 1 included 63 patients, and group 2 included 65 patients. There were no significant differences in sex, age, duration of total GD history, duration of previous drug withdrawal, thyroid size, and thyroid function at drug withdrawal (*P*=0.524, *P*=0.457, *P*=0.078, *P*=0.762, *P*=0.566, *P*=0.069 respectively). The duration of ATD therapy before grouping was not significantly different between groups (*P*=0.687), while the duration of total ATD therapy was statistically different (*P*=0.001).

### Relapse and remission rate

**Figure 1:**
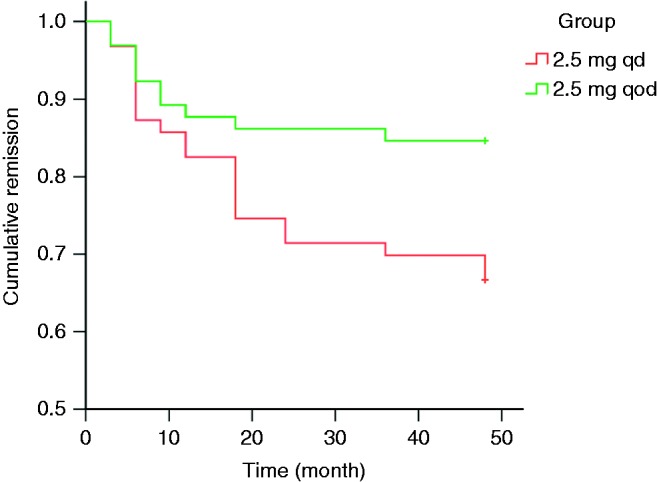
The remission plots of patients (%) remaining euthyroid after drug withdrawal in two groups with different discontinuation drug doses (Kaplan–Meier survival analysis). Log rank test shows *P*=0.024.

The number of cumulative recurrence during the follow-up is given in Table 2. Out of 128, 31 (24.22%) patients had a relapse during the 48 months after MMI withdrawal. Relapse was found in 21 out of 63 (33.33%) in group 1 and ten out of 65 (15.38%) in group 2. In total, 97 out of 128 (75.78%) patients achieved permanent remission at the end of the follow-up. Figure 1 shows that the remission rate of group 2 was significantly higher than that of group 1 (*P*=0.024, Kaplan–Meier survival analysis).

### Relevant factors

**Figure 2:**
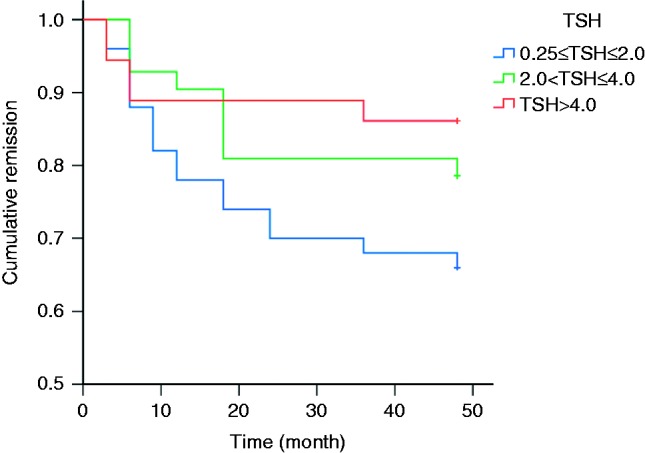
The remission plots of patients (%) remaining euthyroid after drug withdrawal in three groups with different TSH levels at drug withdrawal (Kaplan–Meier survival analysis). Log rank test shows that the *P* values of pairwise comparisons are 0.165 (0.25≤TSH≤2.0 vs 2.0<TSH≤4.0), 0.046 (0.25≤TSH≤2.0 vs TSH>4.0), and 0.444 (2.0<TSH≤4.0 vs TSH>4.0) respectively.

After dividing the 128 enrolled patients into three groups according to the TSH level (reference range: 0.25, 5.0 μIU/ml) at drug withdrawal (group A: 0.25≤TSH≤2.0 μIU/ml, group B: 2.0<TSH≤4.0, group C: TSH>4.0 μIU/ml), it was found that the remission rate of group A was significantly inferior to that of group C (*P*=0.046, Kaplan–Meier survival analysis), and no significant differences were found between any other two groups (group A vs group B, *P*=0.165; group B vs group C, *P*=0.444, Kaplan–Meier survival analysis) (Fig. 2).

All of the enrolled patients were divided by sex, thyroid size at drug withdrawal (0°, I°, and II°), duration of previous drug withdrawal (*t*≤6 months, 6<*t*≤12, 12<*t*≤24, *t*>24 months), and the duration of total ATD therapy (*t*≤12 months, 12<*t*≤24, *t*>24 months) respectively, while no significant differences were found in any grouping (*P*=0.264, *P*=0.526, *P*=549, and *P*=0.200 respectively, Kaplan–Meier survival analysis).

In order to evaluate the prognostic factors, all of the factors (two treatment groups with different discontinuation dose, sex, age, thyroid size, duration of previous drug withdrawal, duration of total ATD therapy, and TSH level at discontinuation) were analyzed together by Cox proportional hazard regression. The results indicated that no significant differences were found in sex, age, thyroid size, duration of previous drug withdrawal, and the duration of total ATD therapy, while significant differences were found in the TSH level and two treatment groups. The hazard ratios, the corresponding 95% CIs, and the *P* values are reported in Table 3.

## Discussion

GD affects ∼0.5% of the population and is the most frequent cause of hyperthyroidism (4, 7, 8). The recurrence rate after ATD therapy is estimated to be 40–60% (2, 3). Most investigators preferred radioactive iodine for these recurrent patients than a second course of ATD therapy (4, 9). It is thought that the ATD therapy will influence the chances of remission, and/or the recurrent patients are unlikely to achieve a remission with a second course of ATD therapy, making radioiodine a better initial therapeutic choice. However, nearly no relevant clinical trials have indicated the poor prognosis of a second course of ATD therapy on recurrent GD.

A hypothesis to explain the remission of GD had been proposed with a simplified model (10, 11). In this simplified model of vicious cycle, hyperthyroidism leads to worsening of the autoimmune aberration, and autoimmune aberration leads to generation of more TSH receptor antibodies and aggravation of the hyperthyroidism. When the patients become euthyroid, the vicious cycle is broken and most patients may experience remission gradually, and it is yet to be approved (12). It has also been proposed that the remission of GD during ATD therapy is linked to restoration of euthyroidism (11, 12). There remains no gold standard for optimal timing of drug withdrawal on GD, despite efforts that many clinical trials have been exploring. Indeed, hyperthyroidism can develop if the dose is not decreased appropriately during ATD therapy, not to mention an inappropriate drug discontinuation. Why will up to 60% patients have a relapse? Perhaps, ATD therapy is not suitable for this patient, but much more probably, it may be related to an inappropriate drug regimen during treatment or improper discontinuation at drug withdrawal. If so, the recurrent GD can still choose the ATD therapy as an initial therapy. One researcher pointed out that factors such as age, sex, and prior history of relapse have not consistently been shown to predict success or failure in individual GD patient management (13). In one previous study, 104 GD patients, who recurred after 18 months' MMI treatment, were randomized into two groups for continuous ATD and radioiodine treatment. The result showed that long-term continuous treatment of hyperthyroidism with MMI is safe, and both the complications and the expense of the MMI treatment do not exceed those of radioactive iodine therapy (14). If that is the case, radioactive iodine therapy may not have potential advantages over ATD even though the first course of ATD therapy has failed.

This study not only recommended the ATD therapy for patients with a relapse, but also explored some indexes for predicting remission. Acceptance of the above mechanism on remission highlights the importance of rendering patients euthyroid and maintaining them in that state for a long period to minimize autoimmune aberration and GD recurrence (11). This may occasionally require more prolonged use of MMI. Meanwhile, the lowest possible dose of the drug should be used to minimize the risk of side effects (11). Several investigators have reported that such therapy may prevent relapse of GD (14, 15, 16, 17). The reason that low-dose MMI therapy plays such a protective effect is that it may decrease the risk of reactivation of the above vicious cycle. Consistent with these reports, the result in this study further confirmed that a much lower discontinuation drug dose of 2.5 mg qod is superior to 2.5 mg qd. Acceptance of the above mechanism on remission also highlights the importance of TSH. Besides, another study concluded that higher TSH levels during, at the end and 3 months after ATD discontinuation were associated with longer duration of remission (18). With a low normal TSH level, the protective effect may not be strong and the vicious cycle is likely to be reactivated under some adverse events (such as bereavement, divorce, and job loss), eventually causing a relapse, while a high or high normal TSH level is much stronger to overcome these events. That may be the mechanism behind the protective effect of high or high normal TSH level in the risk of relapse.

The level of TSH receptor antibody (TRAb) has proven to be a good marker for monitoring the effectiveness of treatment and predicting cases of relapse and remission, especially with the developed functional thyroid-stimulating autoantibodies (TSAb) assay reported in 2012 (19). One limitation of this study is the lack of monitoring of TRAb. This study was started about 10 years ago, and the detection of TRAb was not very accurate at that time. Thus, it was not detected routinely and the recorded information was incomplete. New results with the data of TRAb will be reported in recent studies. We did not study the treatment effect on special populations such as pregnant or lactating women. It has been proved that the *post partum* period is significantly associated with a relapse in GD patients after ATD therapy (20). As the drug of MMI used in this study is not suitable for pregnant and lactating women, all these patients were excluded in this study.

Finally, it is obvious that although much is known about the appropriate use of ATD in the management of GD, this topic still needs some additional clinical research to explore the areas of uncertainty. If GD recurred after drug discontinuation, I would encourage consideration of a second course of drug therapy, although radioiodine therapy or surgery would also be options. It is difficult to confirm in advance as to which patients are likely to experience remission, but a much lower discontinuation drug dose, and high or high normal TSH levels at drug withdrawal are good prognostic signs. However, the above result was based on Chinese patients alone and it needs further confirmation in different ethnic groups.

## Figures and Tables

**Table 1:** Clinical and biological characteristics of the enrolled patients. Reference range of TSH level: 0.25≤TSH≤5.0 μIU/ml.

**Characteristics**	**Group 1** (2.5 mg qd)	**Group 2** (2.5 mg qod)	***P***
Female/male	54/9	53/12	0.524 (*χ* ^2^-test)
Age (mean±s.d.) (range) (years)	34.03±12.49 (7–60)	35.69±12.70 (14–76)	0.457 (Student's *t*-test)
Median duration of total GD history (range) (months)	37.00 (6–204)	48.00 (23–120)	0.078 (Mann–Whitney *U* test)
Median duration of previous drug withdrawal (range) (months)	12.00 (1–120)	12.00 (1–96)	0.762 (Mann–Whitney U test)
Median duration of ATD therapy before grouping (range) (months)	9.00 (3–45)	11.00 (3–85)	0.687 (Mann–Whitney *U* test)
Median duration of total ATD therapy (range) (months)	15.00 (5–51)	20.00 (9–93)	0.001 (Mann–Whitney *U* test)
Thyroid size at drug withdrawal			
0°	23	29	0.566 (*χ* ^2^-test)
I°	35	30	
II°	5	6	
Thyroid function at drug withdrawal (μIU/ml)			
0.25≤TSH≤1.0	9	5	0.069 (*χ* ^2^-test)
1.0<TSH≤2.0	11	25	
2.0<TSH≤3.0	12	16	
3.0<TSH≤4.0	8	6	
4.0<TSH≤5.0	8	5	
TSH>5.0	15	8	

GD, Graves' disease; ATD, antithyroid drug; qd, daily; qod, every other day.

**Table 2:** Number of cumulative recurrences of GD during follow-up.

**Follow-up schedule** (months)	**No. of cumulative relapses** (%)		**No. of overall relapses** (%) (*n*=128)
Group 1 (*n*=63)	Group 2 (*n*=65)
1	0 (0.00%)	0 (0.00%)	0 (0.00%)
3	2 (3.17%)	2 (3.08%)	4 (3.13%)
6	8 (12.70%)	5 (7.69%)	13 (10.16%)
9	9 (14.29%)	7 (10.77%)	16 (12.50%)
12	11 (17.46%)	8 (12.31%)	19 (14.84%)
18	16 (25.40%)	9 (13.85%)	25 (19.53%)
24	18 (28.57%)	9 (13.85%)	27 (21.09%)
36	19 (30.15%)	10 (15.38%)	29 (22.66%)
48	21 (33.33%)	10 (15.38%)	31 (24.22%)

**Table 3:** Multivariate Cox regression analysis for relapse.

**Items**	**HR**	**95% CI**	***P***
Treatment groups: (2.5 mg qod vs 2.5 mg qd)	0.343	0.155, 0.759	0.008
TSH level			
0.25≤2.0	3.256	1.121, 9.456	0.030
2.0≤4.0	1.797	0.593, 5.443	0.300
TSH>4.0	Reference group		
Thyroid size			
0°	Reference group		
I°	0.488	0.219, 1.090	0.080
II°	0.556	0.116, 2.663	0.462
Male vs female	0.467	0.127, 1.721	0.251
Age	0.637	0.275, 1.475	0.292
Duration of total ATD therapy	1.059	0.588, 1.908	0.848
Duration of previous drug withdrawal	1.103	0.799, 1.521	0.552

HR, hazard ratio.
